# Peripheral biomarkers of neuronal damage in neuropsychiatric systemic lupus erythematosus (NPSLE)

**DOI:** 10.3389/fncel.2026.1794050

**Published:** 2026-06-03

**Authors:** María Paulina Reyes-Mata, Aarón González-Palacios, Ariadna López-Llamas, Yeminia Valle, Daniel Ortuño-Sahagún, Miguel Marín-Rosales, Claudia Azucena Palafox-Sánchez

**Affiliations:** 1 Departamento de Disciplinas Filosófico, Metodológicas e Instrumentales, Centro Universitario de Ciencias de la Salud, Universidad de Guadalajara, Guadalajara, Mexico; 2 Grupo de Inmunología Molecular, Centro Universitario de Ciencias de la Salud, Universidad de Guadalajara, Guadalajara, Mexico; 3 Doctorado en Ciencias en Biología Molecular en Medicina, Departamento de Biología Molecular y Genómica, Centro Universitario de Ciencias de la Salud, Universidad de Guadalajara, Guadalajara, Mexico; 4 Laboratorio de Neuroinmunobiología Molecular, Instituto de Neurociencias Traslacionales, Centro Universitario de Ciencias de la Salud, Universidad de Guadalajara, Guadalajara, Mexico; 5 Departamento de Clínicas Médicas, Centro Universitario de Ciencias de la Salud, Instituto de Investigación en Ciencias Biomédicas (IICB), Universidad de Guadalajara, Guadalajara, Mexico

**Keywords:** autoimmune disease, neuropsychiatric systemic lupus erythematosus (NPSLE), brain-specific biomarkers, CSF biomarkers, serum biomarkers, neuroinflammation, neurological damage

## Abstract

Systemic lupus erythematosus (SLE) is a systemic autoimmune disease with heterogeneous clinical presentations, including Neuropsychiatric SLE (NPSLE), which comprises a spectrum of central and peripheral nervous system manifestations attributable to immune-mediated neuronal and glial injury. Currently, diagnosing NPSLE is challenging due to the heterogeneous clinical manifestations and the lack of specific biomarkers. Breakthrough biomarkers are essential for improving diagnostic accuracy, prognostic assessment, and therapeutic monitoring in NPSLE. Serum biomarkers have been thoroughly examined, including inflammatory molecules such as cytokines, chemokines, and autoantibodies; however, these biomarkers are not brain-specific and have also been associated with other clinical domains of SLE. The present review focuses on neuronal and glial damage biomarkers in the context of NPSLE, highlighting their potential utility as diagnostic or prognostic biomarkers, while underscoring the need for further research in this area. Here, we discuss correlations between serum and cerebrospinal fluid (CSF) levels, supporting the use of serum as a minimally invasive surrogate for CNS assessment. Furthermore, findings on serum biomarkers of neurological damage were reviewed to explore their associations with clinical, demographic, and routine laboratory variables, which could provide insights into disease mechanisms. We identified potential biomarkers and highlighted important research gaps that may guide future investigations.

## 1 Introduction

Systemic lupus erythematosus (SLE) is a chronic, multisystemic autoimmune disease characterized by the production of pathogenic autoantibodies, immune complex formation, and variable involvement of multiple organ systems (Pagkopoulou et al., 2025; Tsokos, 2020). The disease results from a loss of T and B-cell tolerance leading to the excessive production of autoantibodies directed against various self-antigens, particularly nucleic acid-containing epitopes such as double-stranded DNA, nucleosomes, and ribonucleoprotein complexes (Pagkopoulou et al., 2025).

The pathophysiology of SLE involves complex interactions between genetic susceptibility, environmental triggers, and dysregulated immune responses. Key mechanisms include defective B-cell tolerance, enhanced T-cell help, and the formation of long-lived autoreactive plasma cells (Arnaud et al., 2024; Pagkopoulou et al., 2025). Nucleic acid-containing antigens including nucleosomes and nuclear proteins that interact with RNA, are significant in driving autoimmune responses through activation of innate immune sensors (Kontaki and Boumpas, 2010).

The heterogeneous clinical presentation of SLE reflects the diversity of autoantibody profiles and the variable involvement of different pathogenic pathways, including type I interferon signaling, complement dysregulation, and Fc gamma receptor-mediated inflammation (Tsokos, 2020). Given its systemic nature, SLE can affect virtually any organ system; the most affected domains include renal, musculoskeletal, mucocutaneous, cardiovascular, hematologic and neuropsychiatric (Ceccarelli et al., 2023; Rice-Canetto et al., 2024; Tsokos, 2020).

The neuropsychiatric complications are challenging to diagnose and treat (Ding et al., 2024) and are categorized as neuropsychiatric systemic lupus erythematosus (NPSLE), which encompasses a wide range of neurological and psychiatric manifestations (Rice-Canetto et al., 2024). The NPSLE manifestations have a heterogeneous prevalence from 5 to 80% (Rayes et al., 2018). These manifestations are broadly categorized by the American College of Rheumatology (ACR) guidelines, which identify 19 distinct conditions, such as headache, seizure disorders, cerebrovascular disease, polyneuropathy, to mention a few (Liang et al., 1999), and an additional six non-ACR conditions commonly discussed in the literature, such as cerebral venous sinus thrombosis, posterior reversible encephalopathy syndrome, isolated optic neuritis, progressive multifocal leukoencephalopathy, and idiopathic intracranial hypertension (Rice-Canetto et al., 2024). Neurological involvement is categorized into Central Nervous System (CNS) and Peripheral Nervous System (PNS) manifestations. CNS involvement can also be divided into focal (specific, such as headache and seizure disorder, to mention a few) and diffuse (generalized, e.g., anxiety and cognitive dysfunction) manifestations (Mizrachi et al., 2022).

Diagnosis of NPSLE is complex because there are no specific diagnostic biomarkers or imaging findings unique to the condition. The diagnosis primarily relies on expert opinion, clinical symptoms, laboratory testing for blood and cerebrospinal fluid, for example, antiphospholipid antibodies (aPL) positivity, neuroimaging [magnetic resonance imaging (MRI)] abnormalities, and the exclusion of other potential non-SLE-related causes (Patel, 2024). Management is equally complex, and is determined by inflammatory or ischemic affection (Wang et al., 2022). Current treatment approaches are drawn from experiences with other lupus subtypes or similar neuropsychiatric disorders. Overall, NPSLE is treated with high-dose corticosteroids and immunomodulators such as cyclophosphamide, hydroxychloroquine, mycophenolate mofetil or azathioprine (Patel, 2024; Rice-Canetto et al., 2024). While immunosuppression and anti-inflammatory drugs form the backbone of NPSLE treatment, the specific regimen is highly individualized based on the type and severity of the neuropsychiatric manifestation, for example, anti-epileptics for seizures (Rice-Canetto et al., 2024; Wang et al., 2022). Antiplatelet or anticoagulant therapy is also necessary to prevent and manage thrombotic events associated with NPSLE, especially in patients with high titers of aPL autoantibodies, cerebrovascular disease, MRI abnormalities, and cardiovascular risk (Patel, 2024).

Biomarkers for NPSLE are an active area of research, offering hope for improving the diagnosis and prognosis of NPSLE patients. Inflammatory-related biomarkers such as cytokines, chemokines and autoantibodies have been extensively studied elsewhere (Lindblom et al., 2022; Manca, 2021; Song and Kono, 2025). In this review, brain-specific biomarkers in serum and cerebrospinal fluid (CSF) were examined, with particular attention to their interrelationships, to support serum testing as a less invasive and more practical option for routine sampling. In addition, evidence on serum markers of neurological injury is reviewed in relation to clinical symptoms and complementary laboratory findings, highlighting notable associations and unresolved questions that warrant further exploration.

To explore neurological damage-specific biomarkers, we reviewed the scientific literature in PubMed and ResearchGate without restriction on publication year. We included only full-text original articles in English that reported on adults (≥18 years) with NPSLE, in which at least one neurological damage-specific biomarker was measured in blood (serum or plasma) and/or cerebrospinal fluid (CSF). All relevant information available to date was considered, using the combined words: “Lupus Vasculitis, Central Nervous System,” “Neuropsychiatric Systemic Lupus Erythematosus,” “Neuropsychiatric lupus,” “biomarker,” “biological marker,” “brain-specific biomarker,” “CNS-biomarker,” “PNS-biomarker.” After acknowledging the specific neurological damage biomarkers studied in NPSLE context, a deeper search was conducted utilizing the biomarker as a keyword along with NPSLE: “Neurofilaments light-chain (NfL),” “Glial Fibrillary Acidic Protein (GFAP),” “S100B protein,” “UCH-L1,” “brain-derived neurotrophic factor (BDNF),” and “Neuron-Specific Enolase (NSE),” soluble triggering receptor expressed on myeloid cells 2 (sTREM2).”We excluded conference abstracts, reviews, *in vitro* studies, antibody-based reports, and those using neuroimaging, gene polymorphisms, or epigenetics. Titles and abstracts were assessed against predefined inclusion criteria, and full texts were retrieved for detailed evaluation. Eligible studies included observational designs (cross-sectional, longitudinal) and meta-analyses involving SLE patients whose neuropsychiatric symptoms were categorized by the 1999 ACR nomenclature, with at least one comparison group such as non-NPSLE patients, healthy controls, or other diseases. Studies lacking NPSLE categorization were excluded. Ultimately, the final selection comprised research on six neurological damage-specific biomarkers, with three biomarkers represented by multiple studies and explored in greater depth.

## 2 Mechanisms of neuronal injury in NPSLE

**FIGURE 1:**
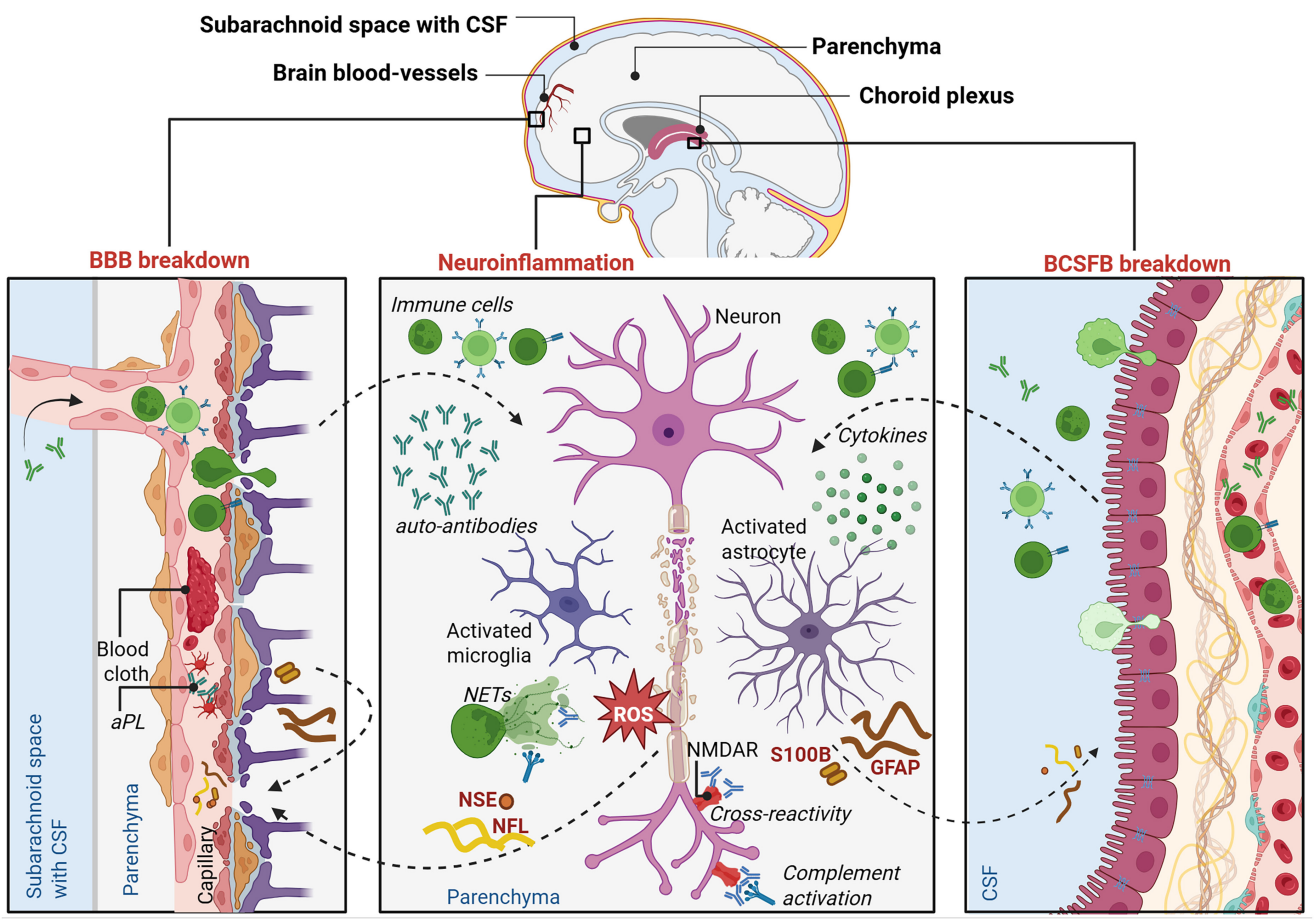
Neuropsychiatric Systemic Lupus Erythematosus (NPSLE) pathophysiology. In NPSLE, neuronal damage arises through a cascade of immune-mediated events that compromise the brain’s protective barriers. The process begins with the breakdown of the blood-brain barrier BBB, where endothelial cells, pericytes, and astrocytic endfeet lose their integrity, allowing immune cells and autoantibodies to infiltrate the brain parenchyma. Blood clot formation associated with aPL leads to ischemic injury. Similarly, breakdown of the BCSFB occurs, allowing the entrance of peripheral inflammatory molecules and cells into nervous system. This breach initiates neuroinflammation, characterized by microglial activation and the release of pro-inflammatory cytokines, thereby disrupting neurotransmitter balance and contributing to neuronal stress. As neurons and glial cells become damaged, they release specific molecular markers indicative of axonal injury or astrocytic damage. These biomarkers enter both the CSF and the bloodstream, facilitated by the concurrent breakdown of the BBB and the BCSFB. CSF, cerebrospinal fluid; BBB, blood-brain barrier; BCSFB, blood-cerebrospinal fluid barrier; aPL, antiphospholipid antibodies; NETs, neutrophil extracellular traps; ROS, reactive oxygen species; NfL, neurofilament light-chain; GFAP, glial fibrillary acidic protein; NMDAR, N-methyl-D-aspartate receptors. Created with BioRender.com.

As a first step in understanding NPSLE, we will explore the mechanisms of neurological injury that lead to glial and neuronal damage and to the release of brain-specific markers into the bloodstream and CSF. Up to now, the limited ability to investigate NPSLE directly in brain tissue, the heterogeneity of clinical manifestations, and the occurrence of neuropsychiatric events independent of lupus make it difficult to understand the pathophysiology, diagnose it, and develop better treatments (Wang et al., 2022). Given the limitations and lack of information, it has been hypothesized that the brain insult in NPSLE can originate from two mechanisms of neurological damage: ischemic and neuroinflammatory pathways, which can occur simultaneously and complementarily (Wang et al., 2022; Figure 1). For example, both inflammatory and thrombotic serum biomarkers are associated with anxiety and depression in NPSLE (Duca et al., 2023; Scherlinger et al., 2021). The mechanisms of nervous system damage include ischemia/thrombosis, mainly associated with antiphospholipid antibodies; IgG antibody and immune complex–mediated neurotoxicity, cytokine-driven inflammation, and brain-resident cell responses, such as microglial activation, all of which are complementary and overlapping (Liu et al., 2022; Ota et al., 2022; Wang et al., 2022).

The ischemic pathway occurs when blood vessels are damaged by intravascular thrombosis. The presence of antiphospholipid antibodies, immunocomplexes, and complement activation in the CNS has been associated with thrombus formation and an increased risk of ischemic stroke (Wang et al., 2022). The mechanisms underlying thrombosis driven by aPL in antiphospholipid syndrome (APS) are complex and multifactorial. They include disruption of the natural anticoagulant and fibrinolytic systems, stimulation of diverse cell populations, such as endothelial cells, monocytes, neutrophils, and platelets, via distinct signaling cascades, and activation of the complement pathway. Together, these alterations establish a pro-thrombotic environment that, when coupled with an additional trigger, such as inflammatory processes, culminates in thrombosis (Schreiber et al., 2018; Yang et al., 2025).

On the other hand, the neuroinflammatory pathway leading to NPSLE could occur when the Blood-Brain Barrier (BBB) is disrupted by the inflammatory context surrounding the highly specialized capillaries that protect the CNS from the periphery (Deijns et al., 2020). This phenomenon can be confirmed by MRI, which demonstrates alterations in cerebral perfusion and BBB permeability in SLE patients with moderate-to-high disease activity, cognitive dysfunction (CD), fatigue, and pain (Salomonsson et al., 2024). Interestingly, BBB dysfunction can be observed independently of neurological involvement (Salomonsson et al., 2023). Biomarkers associated with BBB disruption in NPSLE include elevated CSF albumin levels, which indicate increased barrier permeability (Mizrachi et al., 2022); however, it is not specific to NPSLE. In contrast, microfibrillar-associated protein 4 (MFAP4) is associated with vascular remodeling and is elevated in NPSLE, particularly in patients with CNS involvement (Wegener et al., 2024).

Additionally, BBB dysfunction enables peripheral immune effectors to access CNS targets, including immune cells, cytokines, and autoantibodies that typically do not enter the CNS (Wang et al., 2022). Pathogenic autoantibodies and immune cells can also enter the central nervous system through the Blood-cerebrospinal fluid barrier (BCSFB). Recently, experimental models have hypothesized that BCSFB abnormalities are more critical than BBB abnormalities as a causative factor in NPSLE (Gelb et al., 2018). Later, upon exposure to external immunological factors, resident glia propagate injury through phagocytosis, cytokine production, synaptic stripping, and neurotoxic phenotypes (Nikolopoulos et al., 2021; Sarwar et al., 2021; Wang et al., 2022).

The presence of autoantibodies in the brain induces neuroinflammation. Experimental animal models have demonstrated that antiphospholipid antibodies predominantly cause hyperactivity and cognitive impairment. Conversely, anti-ribosomal P protein (anti-P) antibodies result in depression-like behaviors, cognitive deficits, and olfactory impairments in rats. These findings shed light on the severe neuropsychiatric symptoms observed in clinical cases (Katzav et al., 2014). Moreover, autoantibodies have been shown to bind to neuronal membranes. Serum from patients with SLE and aPL who experience movement disorders has demonstrated elevated levels of IgG that bind to the surface of live neurons. This binding is notably higher than in healthy individuals and in patients with other neurological diseases (Dale et al., 2011). SLE serum, especially from NPSLE patients, contains brain-reactive autoantibodies that are more prevalent and more intense in patients with neuropsyquiatric symptoms. Experimental evidence shows that the autoantibodies primarily target neurons and their nuclei, cytoplasm, and mitochondria, rather than glial cells (Cocco et al., 2023). The formation of antigen-autoantibody complexes induces neuronal apoptosis (Lauvsnes and Omdal, 2012). For example, specific autoantibodies, such as anti-DNA antibodies, have cross-reaction with N-methyl-D-aspartate receptors (NMDARs), inducing non-thrombotic and non-vasculitic abnormalities of the central nervous system (Degiorgio et al., 2001). In the context of NMDARs, the NR2 subunit can be affected by reactive autoantibodies, known as anti-NR2 antibodies. Their presence is linked to disruptions in synaptic transmission and the induction of apoptosis (Hirohata et al., 2014). On the other hand, anti-P antibodies are linked to depression and psychosis by interfering with intracellular calcium and protein synthesis, causing neuronal dysfunction. Another important autoantibody in NPSLE is anti-endothelial cell antibody, which drives vascular inflammation and contributes to cerebral vasculopathy and mood disorders. Anti-ganglioside antibodies may affect peripheral nerves, though their role in NPSLE remains less defined. Together, these antibodies illustrate how immune dysregulation in lupus can directly harm neural structures and impair brain function (Wang et al., 2022). In addition to the direct effects of these autoantibodies, neurotoxicity may impact astrocyte function. *In vitro* experiments have demonstrated decreased expression of Glial Fibrillary Acidic Protein (GFAP) and reduced astrocyte proliferation. These findings suggest a mechanistic link between systemic autoimmunity and astrocyte injury in the context of lupus (Sun et al., 1992).

Moreover, the damage caused by the previously mentioned autoantibodies also leads to immune complex deposition in the neurovasculature, triggering local complement activation, amplifying inflammation, and recruiting myeloid cells that can injure tissue (Liu et al., 2022). Also, the entrance of peripheral immunopathological cells increases the neuroinflammation. For example, neutrophil infiltration contributes significantly to both ischemic and inflammatory pathways observed in NPSLE progression by production of cytokines such as interleukin (IL)-6 and IFN-α, as well as MMP-9, which aids in BBB disruption and inflammation. Also, activation of neutrophil extracellular traps (NETs) serves as an autoantigen reservoir, contributing to the inflammatory loop (Guan et al., 2025).

## 3 Biomarkers for NPSLE in lupus

Neurological involvement in SLE is a frequent and clinically significant manifestation. However, the diagnosis often relies on advanced clinical presentations, by which time signs and symptoms have already progressed considerably (Patel, 2024). Biomarker research in NPSLE is less advanced than in other SLE-affected organs and mainly aims to differentiate NPSLE from similar neurological or psychiatric conditions (Ding et al., 2024; Patel, 2024). Multiple markers have been extensively reviewed in previous publications (Lindblom et al., 2022; Martins de Aquino et al., 2025); however, research on these markers has been delayed, which may be explained by difficulties accessing brain tissue and CSF samples and by heterogeneous results in the serum reflection of nervous system processes.

Most of the research has focused on inflammation-associated biomarkers. In brief, among studies of NPSLE biomarkers, those associated with better outcomes are found in CSF. An example is α-Klotho, a pleiotropic single-pass transmembrane protein that has emerged as a promising diagnostic candidate. Also, Macrophage Colony-Stimulating Factor (M-CSF), which is elevated in Multiple Sclerosis (MS), underscores the neuroinflammatory overlap between these conditions. Other inflammatory molecules found abnormally in CSF, such as IgM, osteopontin, and cytokines IL-17, IL-2, IFN-γ, IL-5, FGF2, and IL-15, have demonstrated excellent diagnostic accuracy (Lindblom et al., 2022). Nowadays, a CSF sample helps rule out infections, and some CSF abnormalities may aid in diagnosing NPSLE, but are not present in all cases (Patel, 2024). Moreover, the CSF sample is invasive and not a routing-based management but rather a suggestion to exclude infections (Castellazzi et al., 2024).

Another research field focuses on serum or plasma biomarkers, which are less invasive. Serum biomarkers have been associated with other organs affected by SLE. For example, cutaneous SLE manifestations have been studied, showing strong correlations with skin involvement and with the prediction of drug effectiveness, including cytokines, immunoglobulins, chemokines, and cell profiles, among others (Ding et al., 2024). Cytokines such as BAFF and APRIL present in serum have been associated with indicators of lupus nephritis (clinical and serological findings) and treatment response. Additionally, chemokines, cell adhesion molecules, and membrane markers have been successfully associated with lupus nephritis (Ding et al., 2024).

Among the most promising diagnostic markers in NPSLE are serum IL-6 and high-mobility group box protein 1 (HMGB1). IL-6 is a cytokine involved in inflammation, T-cell biology, and B-cell antibody production. On the other hand, HMGB1 is a DNA-binding protein that performs various functions in immune and neuronal cells (Lindblom et al., 2022). Conventional biomarkers, such as serum anti-cardiolipin antibodies and low C3 levels, are significantly associated with abnormal MRI findings in NPSLE patients (Tan et al., 2017). Recently, CXCL13 has been associated with active NPSLE. This marker is elevated in active NPSLE patients compared with inactive NPSLE, non-NPSLE, and healthy controls, and correlates with SLE disease activity (Yildirim et al., 2025). However, the above promising markers are also associated with other clinical variables and organic damage in SLE, including the kidney, making them nonspecific to NPSLE (Ayano and Horiuchi, 2023; Liu et al., 2020; Liu et al., 2025; Richter et al., 2025).

Despite efforts, inflammation-associated molecules have shown limited diagnostic performance in NPSLE. Their presence in serum and CSF may reflect systemic immunological activity rather than specific CNS pathology, thus reducing their specificity for neuropsychiatric conditions derived from lupus pathology (Lindblom et al., 2022). For example, preliminary studies of serum BAFF and APRIL have not demonstrated strong associations with neurological involvement, nor have they found correlations between cerebrospinal fluid and serum levels (George-Chandy et al., 2008). BAFF serum concentration is increased in NPSLE patients compared to non-NPSLE patients; however, this increase is also observed in lupus nephritis (Vincent et al., 2013).

Consequently, the search for reliable biomarkers remains an open and pressing area of investigation. A promising direction is to focus beyond inflammatory molecules and seek markers exclusively associated with neurological damage, such as neuron- or glial-cell damage markers or brain-specific autoantibodies. Such specificity would enable the earlier detection of neuropsychiatric manifestations of SLE, thereby minimizing confounding signals from systemic inflammation and potentially improving clinical outcomes through timely intervention. Brain-specific autoantibodies have been extensively studied and reviewed (Cocco et al., 2023; Efthimiou and Blanco, 2009; Manca, 2021). Briefly, anti-GFAP, anti-NMDA, Anti-nucleoporin, and anti-GABA receptor (anti-GABAR) antibodies are brain-specific autoantibodies associated with NPSLE, specific neuropsychiatric manifestations, and imaging abnormalities, suggesting a potential role in the pathophysiology and diagnosis of CNS lupus (Cocco et al., 2023; Lindblom et al., 2022; Sanna et al., 2000). Among neuronal damage biomarkers, a few have been studied in neurological conditions such as Alzheimer’s disease, Parkinson’s disease, and Multiple Sclerosis, among others (Alcolea et al., 2023). The neuron- and astroglia-derived most researched markers for nervous system damage are Neurofilaments light-chain (NfL), GFAP, sphingolipids, p-tau variants, synaptic proteins, Ubiquitin Carboxyl-terminal Hydrolase L1 (UCH-L1), and S100B (Alcolea et al., 2023; Koníčková et al., 2022), also molecules associated with neuron homeostasis, such as brain-derived neurotrophic factor (BDNF) and Neuron-Specific Enolase (NSE) (Ibrahim et al., 2022; Sierakowska et al., 2025). Of these, NfL, GFAP, S100B, UCH-L1, BDNF, and NSE have been studied in NPSLE. Therefore, this review focuses on neuronal and glial damage biomarkers in the context of NPSLE, highlighting their potential utility as diagnostic or prognostic biomarkers, while underscoring the need for further research in this area.

### 3.1 Neurofilament Light-chain (NfL)

#### 3.1.1 NfL as a biomarker for NPSLE

Neurofilaments (NFs) are a unique class of intermediate filaments (IFs) found in neurons, where they provide essential structural support. NFs are composed of subunits; the backbone of the neurofilament is mainly formed by NfL, along with peripherin (in the PNS) or α-internexin (in the CNS), while Neurofilament Middle (NfM) and Neurofilament Heavy (NfH) associate more peripherally (Yuan et al., 2017). When the axonal membrane is disrupted, neurofilaments are released into the interstitial space, CSF, and eventually into the serum (Balajková et al., 2025). Neurofilaments, particularly NfL, have been extensively studied in neuroinflammatory and neurodegenerative diseases, where they are found in high concentrations in blood and CSF (Gaiottino et al., 2013). Moreover, it was detectable in 99.6% of samples from healthy controls, and it is stable over time within individuals (Lanz et al., 2025).

In SLE, studies from almost 40 years ago revealed the detection of anti-neurofilament antibodies in the serum of patients with NPSLE (Kurki et al., 1986; Robbins et al., 1988), and while these studies do not fully elucidate the precise immunological explanation for this increase, they provide insight into the pathophysiology of neurological damage and neurofilaments as a target of neurological damage. Recently, the potential role of neurocytoskeletal structures, especially NfL released by damaged neurons, has been suggested in SLE (Balajková et al., 2025). Neurofilaments, particularly NfL, are considered promising because their presence in bodily fluids reflects neuronal damage (Engel et al., 2021).

Naturally, CSF content reflects neuronal damage-derived molecules, such as NfL, with great success. One study found that CSF NfL levels were 7 times higher in patients with neurologic involvement than in those with non-neurological involvement, and 51 times higher than in healthy controls (Trysberg et al., 2003), suggesting that CSF NfL levels may be a potential biomarker. Actually, CSF NfL levels had a sensitivity of 74%, a specificity of 65%, and a Positive Predictive Value (PPV) of 68%, which is considered a good overall predictive capability (Trysberg et al., 2003). However, the other two recent studies have failed to find a difference in this CSF marker between NPSLE patients and non-NPSLE patients (Balajková et al., 2025; Zervides et al., 2022). Interestingly, one of these two found a difference, but in serum samples (Balajková et al., 2025). Besides, CSF samples are more complex to obtain and raise ethical concerns. Therefore, a peripheral blood sample is a good alternative for detecting NfL and exploring its usefulness in NPSLE. To our knowledge, six studies have evaluated the diagnostic potential of peripheral NfL measurements in patients with NPSLE.

As stated before, after CNS injury, NfL are released into the bloodstream and CSF. In cases of neurological involvement in NPSLE, neurofilaments can leak directly from injured nerves into the circulation, leading to elevated serum NfL levels. Therefore, it is of interest to correlate NfL levels between the two compartments. In fact, a positive correlation has been observed between plasma and CSF NfL concentrations in SLE (Lauvsnes et al., 2022; Zervides et al., 2022), NPSLE (Balajková et al., 2025), and diverse neurological conditions (Gaiottino et al., 2013; Halbgebauer et al., 2025). Given this information, serum NfL measurements offer a promising non-invasive alternative for distinguishing neurological involvement in SLE and warrant further investigation.

Multiple studies have described significantly higher serum NfL levels in patients with NPSLE compared to control groups (Balajková et al., 2025; Kammeyer et al., 2024; Wegener et al., 2024), and compared to non-NPSLE patients (Balajková et al., 2025; Engel et al., 2021; Kammeyer et al., 2024). Also, serum NfL levels have discriminative capacity to differentiate between NPSLE and non-NPSLE patients, with an AUC of 0.646 (Engel et al., 2021), and between active NPSLE and non-active NPSLE with an AUC of 0.96 (Kammeyer et al., 2024). However, Zervides et al. (2022) did not find this difference. In this study, SLE patients were classified as NPSLE using three models with varying levels of stringency, and across all three models, NPSLE and non-NPSLE groups did not differ; both groups had higher serum NfL levels than healthy controls (Zervides et al., 2022), and these findings were confirmed by Wegener et al. (2024). This suggests that nervous system damage in SLE occurs even when it is not clinically evident. This discrepancy could be related to the clinical characteristics of the patient cohorts and explain the heterogeneous findings across studies. Regarding, most of the SLE patients included had low disease activity (Wegener et al., 2024; Zervides et al., 2022). By contrast, in the studies reporting higher serum NfL levels in NPSLE, patients had experienced active major neuropsychiatric events within the previous six months, reflecting active disease (Kammeyer et al., 2024) or most of the patients had SLEDAI-2K score of 10 or higher (Balajková et al., 2025), which could mean that NfL are higher during disease activity. However, such comparative analyses are not feasible in all studies, as disease activity was not assessed (Engel et al., 2021), underscoring the need for further investigations and assessment of disease activity. Table 1 summarizes these findings.

**TABLE 1:** Studies investigating NfL in NPSLE.

Sample (measures of dispersion)	HC			Patients without NPSLE			Patients with NPSLE			Serum		correlation	ROC	References
	*n*	Serum NfL	CSF NfL	*n*	Serum NfL	CSF NfL	*n*	Serum NfL	CSF NfL	NPSLE vs. non-NPSLE	NPSLE vs. HC			
CSF M, SEM	99	n.a.	40, 11 ng/L	68	n.a.	267, 178 ng/L	31	n.a.	2,066, 1,167	**n.a.**	**n.a.**	n.a.	Sen.: 74% Sp.: 65% PPV: 68% NPV: 71%	(Trysberg et al., 2003)
Serum M	n.a.			75	1.06, (log-NFL)	n.a.	69	1.19 (log-NFL)	n.a.	***p* = 0.006**	n.a	n.a.	NPSLE vs. non-NPSLE AUC = 0.646 ***p* = 0.003**	(Engel et al., 2021)
Plasma and CSF	n.a.			5	n.r.	n.r.	62	n.r.	n.r.	Linear regression models (adjusted for age): SLICC/ACR damage index Beta = 0.1 C.I. = 0.039 to 0.16, ***p* = 0.002**		ρ = 0.51, ***p* < 0.001**	n.a.	(Lauvsnes et al., 2022)
Plasma and CSF M (SD)	26	log-NfL pg/mL: 0.71 (0.17)	n.a.	56 49 28	A: 0.83 (0.18) B: 0.83 (0.18) C: 0.81 (0.17)	A: 2.53 (0.27) B: 2.54 (0.30) C: 2.51 (0.28)	16 23 44	A: 0.87 (0.13) B: 0.87 (0.14) C: 0.86 (0.16)	A: 2.73 (0.34) B: 2.65 (0.30) C: 2.62 (0.32)	A: p = 0.40 B:p = 0.33 C:p = 0.17	A: p = 0.003 B: **p < 0.001** C: ***p* < 0.001**	*r* = 0.72, ***p* < 0.001**	n.a.	(Zervides et al., 2022)
Serum or plasma; and CSF M	13	3.6 pg/mL	n.a.	13	4.9 pg/mL	n.a.	13	22.8 pg/mL	3404.9 pg/mL	***p* < 0.001**	Higher in NPSLE, (*p* value n.r.)	Pearson’s *r* = 0.88, ***p* = 0.01**	AUC:0.96, (distinction active major NPSLE) NfL ≥ 6.8 pg/mL, threshold for active major NPSLE	(Kammeyer et al., 2024)
Serum m (IQR)	50	7.4 (5.3, 10.1) pg/mL	n.a.	164	11.9 (7.6, 19.8) pg/mL	n.a.	44	13.0 (8.7, 23.3) pg/mL	n.a.	n.s.	***p* < 0.0001**	n.a.	n.a.	(Wegener et al., 2024)
Serum and CSF M ± SD	31	10.74 ± 4.36 pg/mL	509.7 ± 358.5 pg/mL	16	16.75 ± 12.48 pg/mL	393.4 ± 191.9 pg/mL	32	31.68 ± 36.63 pg/mL	1600 ± 2852 pg/mL	***p* = 0.039**	***p* < 0.001**	r = 0.87 **p < 0.01**	n.a.	(Balajková et al., 2025)

NfL, neurofilament light; HC, healthy controls, NPSLE, neuropsychiatric systemic lupus erythematosus; CSF, cerebrospinal fluid; ROC, receiver operating characteristic analysis; AUC, area under the curve; M, mean; m, median; SD, standard deviation; SEM, standard error of the mean; IQR, interquartile range; bold typography indicates significant p-value; n.s., not significative; n.a., not assessed; n.r., not reported; pg/mL, picograms per milliliter; ng/L, nanograms per liter; log-NFL, logarithmically transformed neurofilament light levels. In Zervides et al. (2022): A, SLICCA model; B, SLICCB model; C, ACR model.

#### 3.1.2 Association of NfL with clinical and laboratory findings

**FIGURE 2:**
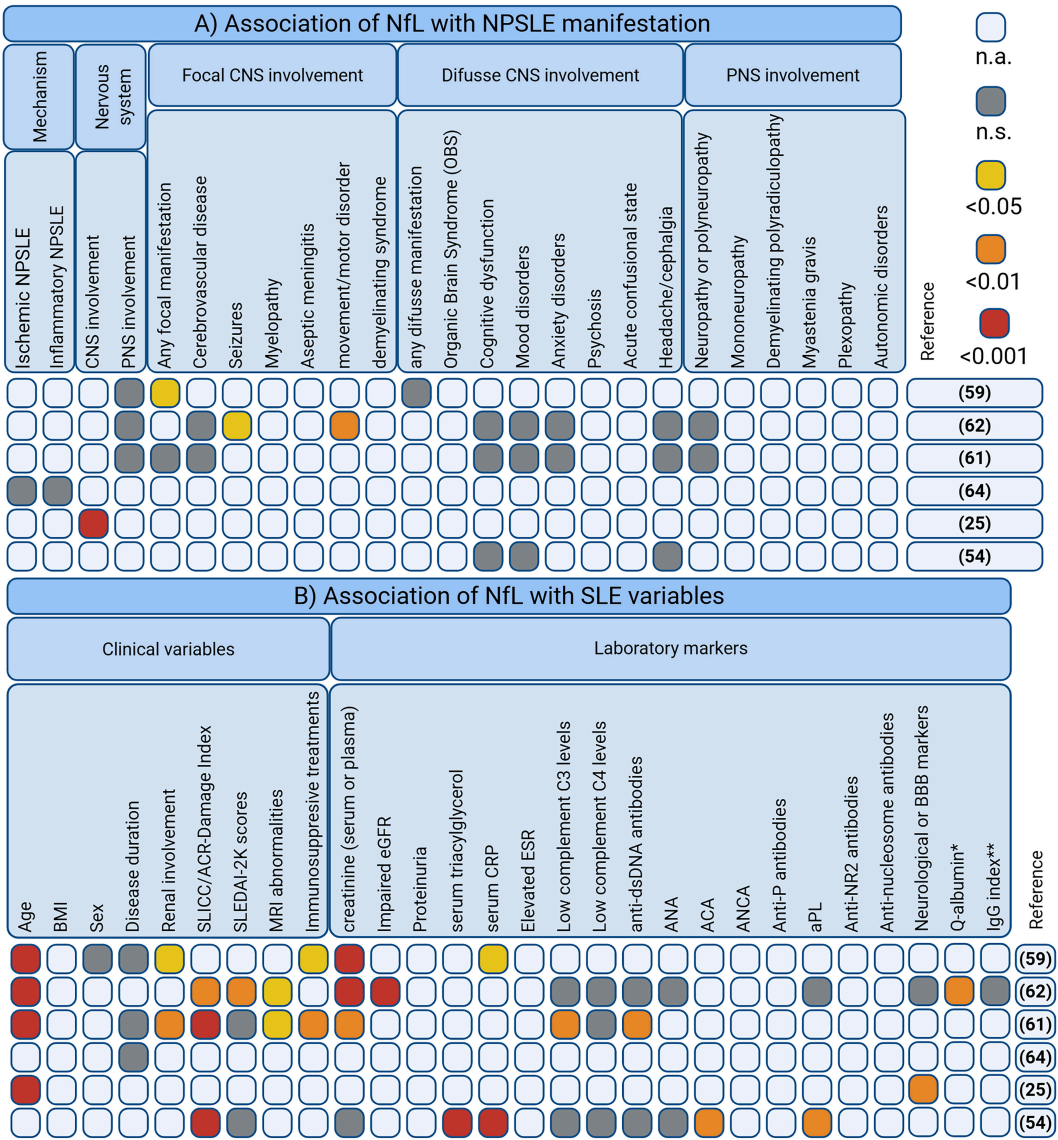
Comparative map of clinical variables related to serum NfL levels. **(A)** Associations of NfL with NPSLE manifestations. **(B)** Associations of NfL with demographic and clinical variables in SLE. This figure provides a comparative overview of how different studies, using diverse statistical approaches, have examined the relationship between serum NfL and clinical variables. Readers can interpret the grid by following the color coding, which indicates statistical significance: Red, *p* < 0.001; Orange, *p* < 0.01; Yellow, *p* < 0.05; Gray, not significant; Blue, not assessed. In this way, the figure highlights both consistently reported associations and underexplored aspects. Further details of each association are described in the main text. Neurofilament light; NPSLE, neuropsychiatric systemic lupus erythematosus; CNS, central nervous system; PNS, peripheral nervous system; BMI, body mass index; SLICC/;ACR, systemic lupus international collaborating clinics/american college of rheumatology; SLEDAI-2K, systemic lupus erythematosus disease activity index 2000; MRI, magnetic resonance imaging; eGFR, estimated glomerular filtration rate; CRP, C-reactive protein; ESR, erythrocyte sedimentation rate; anti-dsDNA antibodies, anti-double-stranded deoxyribonucleic acid antibodies; ANA, antinuclear antibodies; ACA anticentromere antibodies; ANCA, anti-neutrophil cytoplasmic antibodies; Anti-P, anti-ribosomal P; aPL, antiphospholipid antibodies; NfL, neurofilament light chain. Q-albumin, quotient albumin*, is the ratio between CSF/Serum albumin, higher Q-albumin indicates BBB disruption. IgG index** [(CSF/S IgG ratio)/(CSF/S albumin ratio)], higher IgG index indicates IgG CNS production. Created with BioRender.com.

Serum NfL levels have also been associated with NPSLE-specific manifestations, as well as with clinical, demographic, and laboratory SLE markers. Figure 2 summarizes these associations. Wegener et al. (2024) compared serum NfL levels between NPSLE patients with and without CNS involvement, showing that CNS manifestations accounted for the higher NfL concentrations, with a more pronounced difference relative to HC (Wegener et al., 2024). According to this, in Engel et al. (2021), serum NfL levels were higher in patients with focal manifestations of the CNS than in healthy controls. This suggests that focal CNS involvement contributes for most of the elevated serum NfL levels observed in NPSLE patients (Engel et al., 2021). Lauvsnes et al. (2022) in logistic regression analyses found an association of NfL plasma levels and history of seizures and motor disorders, but not with cerebrovascular disease (Lauvsnes et al., 2022), which are focal CNS manifestations. Serum NfL levels have been assessed with diffuse CNS manifestations, such as cerebrovascular diseases, cognitive dysfunction, mood and anxiety disorders, or headache/cephalgia, and none of them have found any association (Balajková et al., 2025; Lauvsnes et al., 2022; Zervides et al., 2022). Consistently, PNS involvement has not been associated with peripheral NfL levels (Engel et al., 2021; Kammeyer et al., 2024; Lauvsnes et al., 2022; Zervides et al., 2022). These studies support evidence that serum NfL level assessment may be most helpful for detecting early focal neuronal damage, whereas it may be less suitable for diagnosing diffuse CNS or PNS neuropsychiatric manifestations. However, in Zervides et al. (2022), plasma NfL were not associated with other CNS focal manifestations (Zervides et al., 2022); this is the only article that failed to find increased serum levels in focal CNS manifestations. Importantly, this study had the lowest sample size of all the studies; therefore, increasing sample size could be an important aspect for generalization, and NfL serum levels are potential biomarkers for these specific clinical manifestations.

Overall, disease activity is assessed using the SLEDAI-2K, and organ damage is evaluated using the SLICC damage Index (SLICC-DI). Both indices were analyzed in relation to serum NfL biomarker studies (Balajková et al., 2025; Lauvsnes et al., 2022; Zervides et al., 2022); out of these, SLICC-DI was associated with serum NfL (Balajková et al., 2025; Lauvsnes et al., 2022; Zervides et al., 2022), while SLEDAI-2K was not (Balajková et al., 2025; Zervides et al., 2022). Balajková et al. (2025) and Zervides et al. (2022) made age-adjusted linear regression analysis and found that organ damage, but not disease activity, was positively associated with serum NfL (Balajková et al., 2025; Zervides et al., 2022). Conversely, Lauvsnes et al. (2022) report an association between SLEDAI and NfL in the linear regression analysis (β = 0.04, *p* = 0.008); however, they showed a weak effect (Lauvsnes et al., 2022). This could indicate that NfL is a biomarker consistently associated with organic damage in SLE. Regarding damage, several reports have found an association between renal impairment and serum NfL levels (Engel et al., 2021; Zervides et al., 2022), also with high serum or plasma creatinine levels and impaired estimated glomerular filtration rate (eGFR) (Engel et al., 2021; Lauvsnes et al., 2022; Zervides et al., 2022). Renal function and CNS impairment have been studied in other contexts (Berry et al., 2022; Hou et al., 2021; Lu et al., 2024; Peng et al., 2024; Tortosa-Carreres et al., 2025), although the causal-effect mechanism has not yet been described. Among the hypothesis stated: kidney function may be affected by excessive NfL filtration; renal impairment could lead to reduced production of neuroprotective molecules, such as erythropoietin and vitamin D; kidney failure could lead to reduced renal clearance of NfL; impaired renal function also predisposes individuals to a higher incidence of cerebrovascular disease, which provokes nervous system damage and NfL release; uremic toxins, such as indoxyl sulfate, can diffuse across the blood-brain barrier and directly damage brain parenchymal cells. This direct damage to neurons and axons would release NfL into circulation. Finally, severe SLE disease is associated with high organ involvement, likely affecting both the kidney and the nervous system simultaneously. Also, comorbidities such as diabetes or hypertension could contribute to organ damage, including the kidneys and the nervous system (Berry et al., 2022; Hou et al., 2021; Lu et al., 2024; Peng et al., 2024; Tortosa-Carreres et al., 2025). Thus, NfL emerges as a promising biomarker reflecting the intertwined pathology of renal and neuropsychiatric involvement in SLE, though mechanistic validation is still needed.

In the CNS, NfL has also been associated with MRI abnormalities. Zervides et al. (2022) report that higher plasma NfL levels were associated with increased total CSF volume (an indicator that may reflect reduced brain volume) (Zervides et al., 2022). Also, Lauvsnes et al. (2022) demonstrated that reduced hippocampal gray matter and corpus callosum white matter volumes were associated with higher plasma NfL levels (Lauvsnes et al., 2022). However, in both studies, the NfL levels did not correlate with white matter lesions (Lauvsnes et al., 2022; Zervides et al., 2022). No other studies had analyzed the association between MRI abnormalities and NfL, making this an important area of research. In summary, NfL appears to be associated with reduced brain volume, highlighting its potential role as a crucial indicator of CNS damage in LES patients.

Laboratory markers such as serum triacylglycerol, serum C-reactive protein (CRP), and low complement C3 and C4 levels have been investigated for association with NfL; among these, serum triacylglycerol has shown a statistically significant association. Authors suggest that triacylglycerol may be associated with accelerated atherosclerosis in SLE, potentially leading to NPSLE (Balajková et al., 2025). Lipid metabolism is disrupted by inflammation, leading to endothelial dysfunction and vascular injury (Reiss et al., 2021), which may also affect the nervous system through an ischemic pathway. Among the other laboratory markers, there is minimal evidence of an association; CRP has been associated with serum NfL levels, underscoring the importance of systemic inflammation in nervous system damage (Balajková et al., 2025; Engel et al., 2021). Overall, low C3 and C4 levels were not associated with serum NfL (Balajková et al., 2025; Lauvsnes et al., 2022; Zervides et al., 2022). This indicates that laboratory markers are under-researched with respect to NfL serum levels, and their clinical utility remains unclear. Among these, triacylglycerol, as a marker of vascular injury, should be further explored to determine whether serum NfL reflects CNS injury, which is probably associated with vascular injury.

Autoantibody production is a hallmark of SLE, and some specific autoantibodies are closely linked to clinical manifestations and subsequent organ damage. Serum autoantibodies have also been associated with levels of NfL, although available data remain limited and inconsistent (Balajková et al., 2025; Lauvsnes et al., 2022; Zervides et al., 2022). An age-adjusted linear regression analysis found no association between anti-dsDNA antibodies and NfL (Balajková et al., 2025); however, another study found a small effect in a multivariate linear regression model between both biomarkers (β = 0.077) (Zervides et al., 2022). On the other hand, an age-adjusted linear regression analysis showed a positive association between serum NfL levels and anti-beta-2-glycoprotein I IgM (Balajková et al., 2025), while another study showed no association between aPL antibody levels and serum NfL (Lauvsnes et al., 2022). Hence, aPL antibodies are important biomarkers of SLE, and their presence has been associated with an increased risk of NPSLE (Bankole et al., 2021). Regarding isotype antibodies, IgG isotypes of lupus anticoagulant (LA) and anticardiolipin antibodies (aCL) have been linked to neuropsychiatric manifestations (Riancho-Zarrabeitia et al., 2021). By deepening the relationship, aPL/NfL could provide insights into their potential clinical use in NPSLE risk assessment. On the other hand, anti-NR2 antibodies were measured in NPSLE patients, but their relationship with serum NfL was not evaluated (Engel et al., 2021), despite prior associations between anti-NR2 antibodies and CSF NfL in SLE patients (Tjensvoll et al., 2020). According to current evidence, while autoantibodies may contribute to NPSLE (Sato et al., 2020), no consistent association has been established with serum NfL, and further studies are required to clarify these relationships. It is plausible that autoantibodies could participate in neurodegenerative processes within the CNS. However, it is not yet possible to determine whether serum NfL levels truly reflect CNS injury potentially driven by autoantibodies, as the indirect association between serum NfL and such injury remains unclear. This highlights the importance of assessing this link at the peripheral level of use in a clinical context.

On the other hand, longitudinal studies have identified serum NfL as a potential biomarker for monitoring clinical outcomes and treatment response in patients with MS (Huss et al., 2020; Mattioli et al., 2020). In NPSLE, information is scarce; only one longitudinal study has analyzed serum NfL levels. This evaluated only 6 patients, and 2 of these with ischemic involvement did not show a reduction in serum NfL (Kammeyer et al., 2024). Stronger evidence is needed to ensure that NfL decreases with therapy. Overall, the evidence suggests that NfL is more strongly associated with focal CNS involvement than with diffuse CNS and PNS involvement, and with organ damage such as renal impairment, supporting its potential as a biomarker for focal CNS injury and organ damage.

### 3.2 Glial fibrillary acidic protein (GFAP)

#### 3.2.1 GFAP as a biomarker for NPSLE

GFAP is an astrocyte-specific intermediate-filament protein that provides structural integrity and mechanical strength to astrocytes (Zheng et al., 2024). Through their GFAP-linked filament network, astrocytes build both a structural backbone and a communication stage (Hol and Pekny, 2015; Zheng et al., 2024). During disease, astrocytes undergo morphological changes to a reactive state called reactive astrogliosis, increasing, among other changes, GFAP expression (Hol and Pekny, 2015). Due to its increased expression during disease, it has been studied as a potential marker of neurological damage, measurable in blood and CSF across a wide range of nervous system diseases, from neuroinflammation and tumors to neurodegeneration and infectious conditions (Zheng et al., 2024). For example, Alzheimer’s Disease, Brain tumors, and cerebral infarction showed higher GFAP levels compared to healthy controls (Kamada et al., 2024). Combined interpretation of GFAP-NfL patterns can help distinguish predominant astrocytic from neuronal injury and has been helpful in other disorders, for example, in Neuromyelitis Optica Spectrum Disorder (NMOSD), where serum GFAP/serum NfL patterns aid relapse diagnostics (Watanabe et al., 2019).

In NPSLE, a few studies have tested GFAP as a candidate biomarker (Table 2). Trysberg et al. (2003) evaluated GFAP in CSF and found it to be three times higher in NPSLE than in SLE patients, and that it decreased after immunological treatment (Trysberg et al., 2003). Interestingly, Kammeyer et al. (2024), demonstrated a strong correlation between CSF and serum GFAP levels, suggesting its potential use as a peripheral marker (Kammeyer et al., 2024); this correlation was observed in a small sample (*n* = 6) and is the only study in NPSLE. In a large cohort of patients with NMOSD and MS (both autoimmune CNS autoinflammatory diseases), the correlation even holds (Watanabe et al., 2019), reinforcing its potential translational value.

**TABLE 2:** Studies investigating GFAP in NPSLE.

Sample (measures of dispersion)	HC			Patients without NPSLE			Patients with NPSLE			Serum		Correlation.	ROC	References
	*n*	Serum GFAP	CSF GFAP	*n*	Serum GFAP	CSF GFAP	*n*	Serum GFAP	CSF GFAP	NPSLE vs. non-NPSLE	NPSLE vs. HC			
CSF M ± SEM	99	n.a.	Males (436 ± 152 ng/L female (387 ± 194 ng/L)	68	n.a.	534 ± 234 ng/L	31	n.a.	1,904 ± 975 ng/L	n.a.	n.a.	n.a.	in CSF: Sensitivity: 48% Specificity: 87% PPV: 79% NPV: 63%	(Trysberg et al., 2003)
Serum or plasma, and CSF M	13	50.4 pg/ml	n.a.	13	51.6 pg/mL	n.a.	13	54.8	20906.8 pg/mL	***p* < 0.001**	Higher in NPSLE, (p-valuen n.r.)	*r* = 0.81, ***p* = 0.03,** (7 patients)	AUC = 0.77 (active major NPSLE vs. non-NPSLE) GFAP ≥ 70.7 pg/mL, threshold for active major NPSLE	(Kammeyer et al., 2024)
Serum m (IQR)	50	67.3 (47.5, 95.5) pg/mL	n.a.	164	102.7 (73.487, 160.441) pg/mL	n.a.	44	118.8 (78.2, 163.4) pg/mL	n.a.	n.s.	***p* < 0.0001**	n.a.	n.a.	(Wegener et al., 2024)

GFAP, glial fibrillary acidic protein; HC, healthy controls., NPSLE, neuropsychiatric systemic lupus erythematosus; CSF, cerebrospinal fluid; ROC, receiver operating characteristic analysis; AUC, area under the curve; M, mean; m, median; SD, standard deviation; SEM, standard error of the mean; IQR, interquartile range; bold typography indicates significant *p*-value; n.s., not significative; n.a., not assessed, n.r., not reported; PPV, positive predictive value; NPV, negative predictive value; pg/mL, picograms per milliliter; ng/mL, nanograms per milliliter.

Kammeyer et al. (2024) report a higher serum concentration of GFAP in patients with active major (severe symptoms) of NPSLE compared with disease-matched SLE controls without active major NPSLE; the study reported an average difference of +3.2 pg/mL (95% CI 1.9–5.5; *p* < 0.001) for GFAP, with reductions after immunotherapy in a small subset of patients (*n* = 6). In the same study, active major NPSLE was associated with concurrent increases in blood GFAP and NfL. Showing acceptable and excellent performance for GFAP (AUC of 0.77), NfL (AUC of 0.96), respectively (Kammeyer et al., 2024). In contrast, Wegener et al. (2024), in a larger SLE cohort, observed higher serum GFAP levels in the non-SLE and NPSLE groups compared with healthy controls, with no difference between NPSLE and non-NPSLE patients. Both GFAP and NfL serum levels in both NPSLE and non-NPSLE were associated with Microfibrillar-associated Protein 4 (MFAP4), the central molecule of this study. MFAP4 was also correlated with other markers of BBB disruption in all the SLE patients. Therefore, the authors concluded that brain damage occurs in SLE independently of clear nervous system manifestations (Wegener et al., 2024). As noted for NfL, patients in the study by Kammeyer exhibited overall high disease activity as active major NPSLE patients were included, whereas low activity predominated in Wegener’s cohort. Accordingly, GFAP levels appear to increase during active disease and decline in inactive states. Importantly, in both studies, the control groups showed lower biomarker levels, reinforcing the evidence of brain damage in SLE. None of the above studies performed a multivariate analysis to assess the combination of the biomarkers. In other neurological diseases, the combination of NfL and GFAP has shown improved predictive value (Fang et al., 2024; Thebault et al., 2023). In summary, there is very little information about GFAP, and studies are contradictory about whether it can serve as an NPSLE biomarker, cohorts’ heterogeneity might be a source of bias in interpretation. Overall, NfL appears to be a stronger diagnostic or predictive biomarker than GFAP. However, the combination of both, along with other markers, could improve NPSLE follow-up, and further studies are required, so the potential of GFAP as biomarker for NPSLE remains.

#### 3.2.2 Association of GFAP with clinical and laboratory findings

**FIGURE 3:**
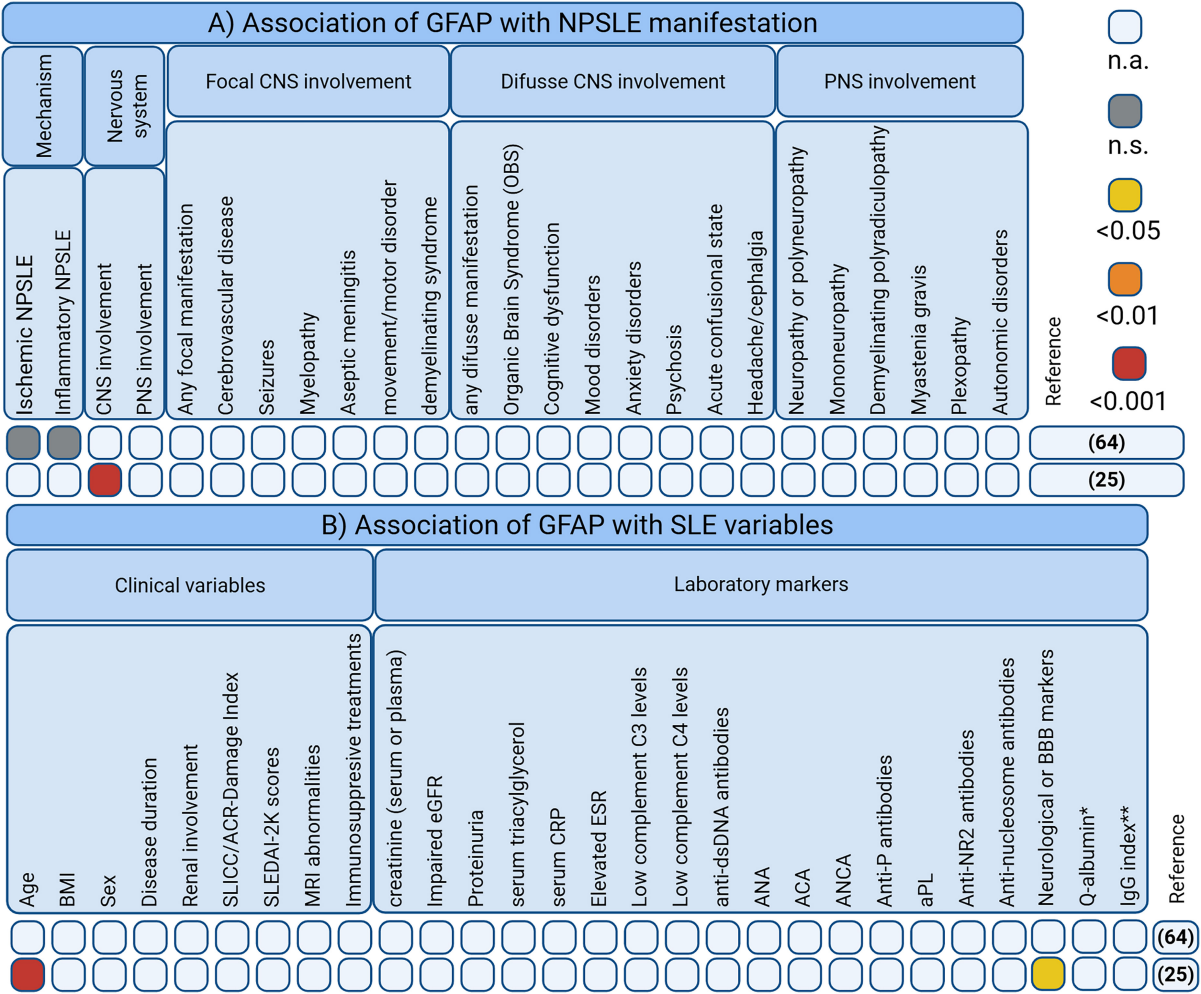
Comparative map of clinical variables related to GFAP serum levels. **(A)** Association of GFAP with NPSLE manifestation and **(B)** Association of GFAP with SLE demographic and clinical variables. This figure provides a comparative overview of how different studies, using diverse statistical approaches, have examined the relationship between serum GFAP and clinical variables. Readers can interpret the grid by following the color coding, which indicates statistical significance: Red, *p* < 0.001; Orange, *p* < 0.01; Yellow, *p* < 0.05; Gray, not significant; Blue, not assessed. In this way, the figure highlights both consistently reported associations and underexplored aspects. Further details of each association are described in the main text. GFAP, glial fibrillary acidic protein; NPSLE, neuropsychiatric systemic lupus erythematosus; CNS, central nervous system; PNS, peripheral nervous system; BMI, body mass index; SLICC/; ACR, systemic lupus international collaborating clinics/american college of rheumatology; SLEDAI-2K, systemic lupus erythematosus disease activity index 2000; MRI, magnetic resonance imaging; eGFR, estimated glomerular filtration rate; CRP, C-reactive Protein; ESR, erythrocyte sedimentation Rate; anti-dsDNA antibodies, anti-double-stranded deoxyribonucleic acid antibodies; ANA, antinuclear antibodies; ACA anticentromere antibodies; ANCA, anti-neutrophil cytoplasmic antibodies; Anti-P, anti-ribosomal P; aPL, antiphospholipid antibodies. Q-albumin, quotient albumin*, is the ratio between CSF/Serum albumin, higher Q-albumin indicates BBB disruption. IgG index** [(CSF/S IgG ratio)/(CSF/S albumin ratio)], higher IgG index indicates IgG CNS production. Created with BioRender.com.

Serum GFAP levels have not been extensively studied in relation to NPSLE-specific manifestations or to clinical, demographic, and laboratory markers of SLE. Figure 3 summarizes and provides evidence for the need to examine associations with other variables. Among these, Wegener et al. (2024) found that patients with CNS involvement had higher GFAP levels (also for NfL) than non-NPSLE patients, suggesting that these molecules are closely associated with their CNS manifestations. However, the direct comparison between PNS and CNS was not reported (Wegener et al., 2024). Kammeyer et al. (2024) failed to find an association of GFAP with CNS or PNS involvement (Kammeyer et al., 2024). Moreover, no other study has attempted to identify these associations. Also, in Wegener et al. work, GFAP was associated with age only in the NPSLE group; finally, GFAP was associated with MFAP4, which is proposed as a BBB disruption marker (Wegener et al., 2024). As shown in Figure 3, the blue squares across the two studies analyzed indicate that most clinical and laboratory variables were not evaluated with serum GFAP. This reflects both the absence of statistical analyses and the lack of laboratory markers consistently quantified across studies.

### 3.3 S100B protein

#### 3.3.1 S100B as a biomarker for NPSLE

S100B is a calcium-binding protein primarily expressed by astrocytes and is also found in other glial cells and some peripheral tissues. S100B participates in a broad spectrum of processes, from providing intracellular scaffolding and regulating cell growth to orchestrating intricate extracellular signaling pathways. Depending on its concentration, S100B can either support neural integrity (e.g., neurotrophic factor production) or drive pathological mechanisms (e.g., increased oxidative stress), playing a pivotal role in both normal brain function and the progression of neuroinflammation and neurodegenerative conditions (Langeh and Singh, 2021). The protein S100B has been widely studied as a biomarker across numerous neurological and psychiatric disorders, with better outcomes in acute than in chronic states (Abboud et al., 2023). In traumatic brain injury (TBI), it is the only single biomarker with a validated clinical cutoff for mild cases (Rogan et al., 2022). In other acute diseases, such as subarachnoid hemorrhage, ischemic stroke, epilepsy, and migraine, elevated levels correlate with severity and outcomes (Abboud et al., 2023). It has also been linked to Alzheimer’s, Parkinson’s, MS, schizophrenia, affective disorders, and others (Langeh and Singh, 2021; Rogan et al., 2022).

A few studies have assessed serum and CSF S100 B levels in patients with NPSLE (Table 3). Of these, Lauvsnes et al. (2018) sampled CSF exclusively and compared levels between the entire SLE group (in which 92% of patients had neurological manifestations) and Sjögren’s Disease (SD). Therefore, NPSLE versus non-NPSLE comparisons were not performed, the specific levels were not reported, and healthy controls were not included. However, it is interesting to note that S100B was associated with TWEAK, a TNF superfamily member, and was also related to increased BBB permeability in both SLE and SD patients, suggesting its involvement in intracerebral processes and BBB integrity (Lauvsnes et al., 2018). Another study measured S100B in serum and CSF in NPSLE patients. Even when a correlation analysis was not performed, there were parallel changes in serum and CSF S100B levels, including a decrease in six NPSLE patients after 3 months of standardized treatment, suggesting an underlying relationship between both compartments (Yang et al., 2008). The only study, to our knowledge, that assessed both CSF and serum across all groups was Fragoso-Loyo et al. (2010), who found no significant correlation between serum and CSF S100B in SLE patients, suggesting that serum may not reflect CNS damage (Fragoso-Loyo et al., 2010). It would be of interest to perform this analysis on a larger sample and in the NPSLE and non-NPSLE subsets. In a study of TBI, serum and CSF S100B concentrations were highly heterogeneous over time and individuals; however, when the ratio (to assess CSF drainage into the blood) was calculated, a correlation with clinical variables was observed (Kleindienst et al., 2010). This means that while CSF and serum might not be consistently correlated, their ratio could provide information about BBB stability and correlate with clinical outcomes.

**TABLE 3:** Studies investigating S100B in NPSLE.

Sample (measures of dispersion)	HC			Patients without NPSLE			Patients with NPSLE			Serum		Correlation	ROC (AUC)	References
	n	Serum S100B	CSF S100B	n	Serum S100B	CSF S100B	n	Serum S100B	CSF S100B	NPSLE vs. non-NPSLE	NPSLE vs. HC			
Serum m (IQR)	15	0.11 (0.04 to 0.16) (ng/mL)	n.a.	26	iSLE (*n* = 13) (0.21 [0.13 to 0.26]) aSLE (*n* = 13) (0.16 [0.12 to 0.26]) (ng/mL)	n.a.	6	(0.41 [0.39–0.65]) (ng/mL)	n.a.	***p* < 0.005**	***P* < 0.0001**	n.a.	n.a.	(Portela et al., 2002)
Serum m (IQR)	25	0.088 (0.013–0.124) (ng/mL)	n.a.	64	0.062 (0.026–0.109) (ng/mL)	n.a.	23	0.164 (0.113–0.332) (ng/mL)	n.a.	***p* < 0.01**	***p* < 0.001**	n.a.	NPSLE (AUC = 0.77, 0.65 to 0.89). aNPSLE (0.82, 0.71–0.93).	(Schenatto et al., 2006)
Serum, CSF only NPSLE. M ± SD	20	0.103 ± 0.065 μg/l	0.114 ± 0.014 μg/l	92	0.110 ± 0.091 μg/l	n.a.	65	0.179 ± 0.095 μg/l	OBS: 0.289 ± 0.073 μg/l Seizures: 0.267 ± 0.125 CVA: 0.288 ± 0.092 Psychosis: 0.258 ± 0.120	***p* < 0.001**	***p* = 0.005**	n.a.	n.a.	(Yang et al., 2008)
Serum and CSF m (min-max)	18	Primary NP (*n* = 4): 24.9 (16.4–38.7) Nonauto- immune (*n* = 14): 24.4 (16.3–42.4)(μg/l)	Primary NP (*n* = 4): 156.2 (46.6–1952) Nonauto- immune (*n* = 14): 208.9 (37.1–485.9) (μg/l)	17	SLE Surgical (*n* = 13): 32.1 (22.6–111.7) SLEsm (*n* = 4): 29.8 (17.3–293)(μg/l)	SLE Surgical (*n* = 13): 149.7 (108.7–393.2) SLEsm (n = 4): 1706.8 (215.3–2500)(μg/l)	40	cNPSLE (*n* = 36): 25.9 (16.3–479.1) pNPSLE (*n* = 4): 25.8 (20.3–37.9) (μg/l)	cNPSLE (*n* = 36): 177 (17.1–2500) pNPSLE (*n* = 4): 121.3 (86.7–182.4) (μg/l)	No differences were found between any of the studied groups		*r* = 0.23 (*p* = 0.08)	Threshold at 3 SD above control: Sensitivity 20%, Specificity 65%, Accuracy 33%.	(Fragoso-Loyo et al., 2010)
Serum m (IQR)	20	0.019 ng/mL; 0.013–0.023	n.a.	12	0.017 ng/nL; 0.013–0.030	na	35	0.031 ng/mL; 0.017–0.042	n.a.	n.s.	***p* = 0.0081**	n.a.	polyneuropathy AUC = 0.742, ***p* = 0.021**	(Noris-García et al., 2018)
CSF m (ranges)	n.a.	n.a.	n.a.	4	n.a.	SLE group (*n* = 50): 221 (110–420) pg/mL	46	na	NP symptoms in 92% SLE (levels n.r.)	n.a.	n.a.	n.a.	n.a.	(Lauvsnes et al., 2018)
Serum M	39	9.6 pg/mL	n.a.	n.a	n.a.	n.a.	35	16.5 pg/mL	n.a.	n.a.	n.s.	n.a.	n.a.	(Jasiak-Zatoñska et al., 2022)

HC, healthy controls, NPSLE, neuropsychiatric systemic lupus erythematosus; CSF, cerebrospinal fluid; ROC, receiver operating characteristic analysis; AUC, area under the curve; M, mean; m, median; SD, standard deviation; IQR, interquartile range; min, minimum value; max., maximum value; bold typography indicates significant *p*-value; n.s., not significative; n.a., not assessed; n.r., not reported; μg/l, microgram per milliliter; pg/mL, picograms per milliliter; ng/mL, nanograms per milliliter; NP, neuropsychiatric; aNPSLE, acute NPSLE; SLEsm, SLE patients with septic meningitis; cNPSLE, central nervous system NPSLE patients; pNPSLE, peripheral nervous system NPSLE patients; iSLE, inactive SLE; aSLE, active SLE.

Beyond the CSF-serum comparison, identifying an association between S100B and NPSLE in serum could make S100B a less invasive biomarker (Jasiak-Zatoñska et al., 2022; Noris-García et al., 2018; Portela et al., 2002; Schenatto et al., 2006). In the Jasiak-Zatoñska et al. (2022) study, which included patients with NPSLE, NMOSD, and MS, serum S100B was detectable in 9 of 35 patients with NPSLE (25.7%). Among these nine patients, S100B levels were elevated relative to healthy controls; however, MS patients had the highest S100B levels. Interestingly, only 20% of healthy controls and none of the NMOSD patients had detectable S100B (Jasiak-Zatoñska et al., 2022). These results could lead one to believe that S100B is not a good biomarker due to its low detectability in most samples. However, in meta-analyses of other neurological diseases, S100B missed detection is not reported (Rogan et al., 2022; Yang et al., 2008; Zheng et al., 2020), and in the studies included here for NPSLE, no other study reported S100B undetected; only in the Portela et al. (2002) study, a few controls had undetected S100B (observed in graphics) (Portela et al., 2002).

In NPSLE, S100B has been consistently elevated. Noris-García et al. (2018) found that NPSLE patients have higher levels than non-NPSLE patients, with an acceptable performance (AUC of 0.706) (Noris-García et al., 2018). Schenatto et al. (2006) reported similar findings regarding diagnostic accuracy, with comparable results for NPSLE (AUC of 0.77) and improved performance in acute NPSLE (AUC of 0.82) (Schenatto et al., 2006). In the same line, in the Yang et al. (2008) study, serum S100B levels were higher in NPSLE patients than in non-NPSLE patients and controls. Non-NPSLE patients and healthy controls were not different. This is interesting because S100B seems to rise only when neurologic manifestations are present (Yang et al., 2008). Also, in Portela et al. (2002) study, NPSLE patients had higher levels than active and inactive SLE, which in turn had higher levels than controls (Portela et al., 2002). However, in Fragoso-Loyo et al. (2010) study, no differences were observed among groups. It is important to note that the control groups consisted of patients undergoing elective surgery and those diagnosed with primary neuropsychiatric syndromes. Therefore, they were not entirely healthy, and other underlying pathophysiological mechanisms may have influenced the observed S100B levels (Fragoso-Loyo et al., 2010). In conclusion, these findings suggest that S100B is a promising candidate for further investigation as a biomarker. However, variability in study results and possible technical limitations suggest the need for standardized methods to improve S100B detection.

#### 3.3.2 Association of S100B with clinical and laboratory findings

**FIGURE 4:**
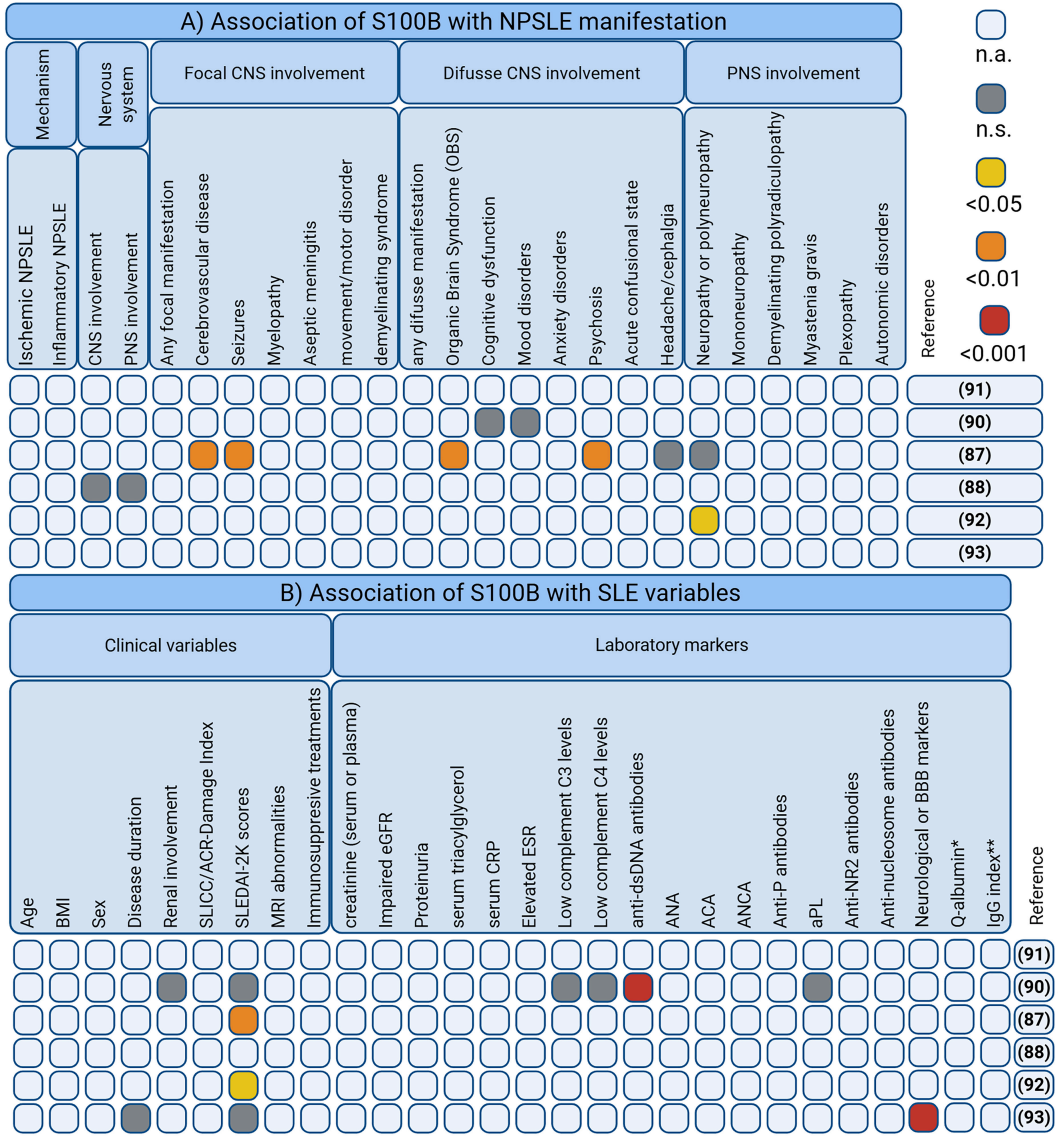
Comparative map of clinical variables related to S100B serum levels. **(A)** Association of S100B with NPSLE manifestation and **(B)** Association of S100B with SLE demographic and clinical variables. This figure provides a comparative overview of how different studies, using diverse statistical approaches, have examined the relationship between serum S100B and clinical variables. Readers can interpret the grid by following the color coding, which indicates statistical significance: Red, *p* < 0.001; Orange, *p* < 0.01; Yellow, *p* < 0.05; Gray, not significant; Blue, not assessed. In this way, the figure highlights both consistently reported associations and underexplored aspects. Further details of each association are described in the main text. NPSLE, neuropsychiatric systemic lupus erythematosus; CNS, central nervous system; PNS, peripheral nervous system; BMI, body mass index; SLICC/;ACR, systemic lupus international collaborating clinics/american college of rheumatology; SLEDAI-2K, systemic lupus erythematosus disease activity index 2000; MRI, magnetic resonance imaging; eGFR, estimated glomerular filtration rate; CRP, C-reactive protein; ESR, erythrocyte sedimentation rate; anti-dsDNA antibodies, anti-double-stranded deoxyribonucleic acid antibodies; ANA, antinuclear antibodies; ACA, anticentromere antibodies; ANCA, anti-neutrophil cytoplasmic antibodies; Anti-P, anti-ribosomal P; aPL, antiphospholipid antibodies. Q-albumin, quotient albumin*, is the ratio between CSF/Serum albumin, higher Q-albumin indicates BBB disruption. IgG index** [(CSF/S IgG ratio)/(CSF/S albumin ratio)], higher IgG index indicates IgG CNS production. Created with BioRender.com.

As observed in Figure 4, there is limited information on the correlation between S100B and clinical and laboratory markers, with very little performance across these variables. In summary, Yang et al. (2008) found that S100B was higher in the NPSLE group than in controls. However, this difference was mainly observed in cerebrovascular disease, organic brain syndrome, and seizures, suggesting that these manifestations may be more strongly associated with S100B levels (Yang et al., 2008). Also, ROC curve analysis found that S100B helped distinguish peripheral neuropathy among patients (Noris-García et al., 2018). However, other studies found no association with cognitive dysfunction, mood disorders, or headache (Schenatto et al., 2006; Yang et al., 2008). Also, Fragoso-Loyo et al. (2010) found that serum S100B levels do not differentiate SLE patients with central neurological manifestations from those with peripheral neurological manifestations (Fragoso-Loyo et al., 2010). It seems that S100B cannot differentiate between clinical manifestations in NPSLE. Given that there are not enough studies to validate these findings, this topic warrants further exploration. Regarding global disease activity in SLE, two studies found no associations between serum S100B and SLEDAI-2K scores (Jasiak-Zatoñska et al., 2022; Schenatto et al., 2006), nor at baseline or follow-up (Jasiak-Zatoñska et al., 2022). Yang et al. (2008) reported a decrease of S100B levels in six NPSLE patients after three months of treatment, indirectly associated with improvement (Yang et al., 2008). In the work of Noris-García et al. (2018), S100B levels were generally higher in active SLE patients than in controls, suggesting an association between SLEDAI and S100B. However, no differences were found between active and inactive SLE patients (Noris-García et al., 2018). In conclusion, disease activity, as stated by SLEDAI-2K scores, is not clearly associated with S100B. No other clinical variable was assessed or found to correlate significantly.

In NPSLE patients, autoantibodies and complement components C3 and C4 were measured. Patients with positive anti-dsDNA antibodies had significantly higher serum S100B levels than those without these antibodies. In contrast, the other laboratory markers were not associated with S100B (Schenatto et al., 2006). Poor information is available regarding S100B and clinical and laboratory variables. In some studies, even when organ and tissue involvement and specific NPSLE manifestations were described, these variables were not associated with S100B levels (Fragoso-Loyo et al., 2010; Yang et al., 2008). Further studies must focus their efforts on this matter.

In summary, S100B appears to be a promising biomarker for diagnosing and monitoring NPSLE. Although its correlation with other key clinical and confounding variables is unclear, it remains an important area for investigation. Moreover, the search for panels and ratios with other biomarkers might improve S100B performance as a diagnostic or prognostic biomarker.

### 3.4 Other neurological damage markers

Apart from the molecules already mentioned, for which more than one article has been published in NPSLE patient populations, other molecules have also been explored. Although the available information is limited, these findings may serve as a starting point for deeper investigation. Among them are the proteins UCH-L1, BDNF, NSE, and sTREM2, each of which is discussed individually below.

UCH-L1, also known as PGP9.5, is an abundant neuron-specific deubiquitinating enzyme with little expression in other tissues that plays a crucial role in maintaining protein homeostasis in the nervous system and is essential for axonal integrity (Bishop et al., 2016). Recent studies have highlighted the potential role of UCH-L1 in neurodegenerative diseases like Parkinson’s disease, Alzheimer’s disease, and brain injury (Mi and Graham, 2023). In NPSLE, UCH-L1 autoantibodies were quantified in CSF to diagnose and assess disease severity, providing new insights into its pathogenesis and potential therapeutic targets (Guo et al., 2022; Li et al., 2019). UCH-L1 levels in CSF are significantly elevated in patients with severe NPSLE compared with those with mild NPSLE and SLE controls, and correlate with disease activity and the number of neuropsychiatric manifestations (Li et al., 2019). However, with a multiplex assay, UCHL-1 was not detectable in the serum of NPSLE or non-NPSLE patients, detectable only in CSF (Li et al., 2019). Inconsistently, studies that include healthy controls, glioma, and TBI subjects, UCHL-1 has been detected by ELISA in the serum (Ramezani et al., 2018; Zhu et al., 2023). Therefore, further studies on NPSLE should be conducted, paying careful attention to methodological details.

BDNF is a neurotrophic protein produced by both neurons and glial cells with a critical role in neuronal survival, maintenance, synaptic plasticity, and regulation of neurotransmission. Reduced levels of BDNF in the brain and serum have been linked to various neurological disorders, making it a promising biomarker for disease progression and therapeutic response (Lima Giacobbo et al., 2018). In NPSLE, a recent meta-analysis analyzed eight studies. This systematic review and meta-analysis concluded that there is no significant association between blood BDNF levels and SLE. The observed heterogeneity among studies suggests that factors such as sample size, age, and sex may influence BDNF levels in SLE patients, highlighting the need for further high-quality research to clarify BDNF’s role across SLE subgroups and its relevance as a biomarker (Shobeiri et al., 2023).

NSE is a specialized form of the glycolytic enzyme enolase, essential for generating ATP and NADH that fuel cellular metabolism. Because of its role in neuronal activity, NSE has been widely investigated as a diagnostic and prognostic biomarker in numerous neurological and psychiatric disorders (Sierakowska et al., 2025). To our knowledge, one study has measured serum NSE levels in the context of NPSLE. In Hawro et al. (2015) study, patients classified as NPSLE were included, and NSE was lower than in non-NPSLE patients and healthy controls. Also, inverse correlations between NSE and disease activity, as well as the number of neuropsychiatric manifestations, were observed in NPSLE. They evaluated the best cut-off value with a sensitivity of 76.0% and a specificity of 63.6% for predicting NPSLE. Authors claim that the chronic damage state of NPSLE leads to reduced overall NSE synthesis and/or concentration in the bloodstream, thereby mirroring a sustained decline in neuronal function and tissue metabolism (Hawro et al., 2015). This article serves as a first step toward evaluating NSE as a neurological damage-specific marker in NPSLE, with the potential to detect chronic damage in these patients.

Wang et al. (2025) investigated serum and CSF sTREM2 as potential biomarkers for neuropsychiatric manifestations in SLE (Wang et al., 2025). TREM2 is a microglial surface receptor, and its soluble form (sTREM2) reflects microglial activation (Liao et al., 2025). Elevated sTREM2 levels in CSF and plasma have been associated with diverse neuroinflammatory conditions, although its precise role remains unclear (Filipello et al., 2022; Liao et al., 2025). In their study, patients were classified as NPSLE according to ACR criteria, and a predictive nomogram was developed integrating disease activity, organ damage, coagulation parameters, immune cell profiles, and serum sTREM2. This model demonstrated strong discriminative capacity, with high accuracy and predictive performance in both the training and validation cohorts (AUCs of 0.914 and 0.842, respectively). Interestingly, sTREM2 also decreased in serum after treatment and the resolution of neuropsychiatric symptoms in 16 NPSLE patients (Wang et al., 2025). Therefore, sTREM2 is a promising biomarker for NPSLE diagnosis and prognosis and could be used alongside other variables to predict the development of neuropsychiatric manifestations.

Finally, in other neurodegenerative diseases, sphingolipids, p-tau variants, and synaptic proteins have shown good performance as indicators of neurodegeneration (Agnello et al., 2025; Alcolea et al., 2023; Koníčková et al., 2022; Mazzucchi et al., 2020). These biomarkers, though not yet validated in NPSLE, are biologically relevant given the disease’s core mechanisms (neuroinflammation, BBB disruption, and immune-mediated neuronal damage) and are promising candidates for future clinical and translational studies.

## 4 Current challenges and study limitations in neurological damage-specific biomarkers

The evaluation of biomarkers associated with neurological damage in NPSLE is a relevant topic with significant potential to improve patients’ diagnosis and management. However, the current search has considerable methodological heterogeneity, complicating the comparability of findings across studies.

Differences in NPSLE classification frameworks are important. Some groups adhered strictly to ACR criteria, while others employed ACR plus modified or consensus approaches, such as the Bortoluzzi algorithm (Balajková et al., 2025), alternative CNS-focused definitions (Trysberg et al., 2003) based on the SLICC-DI and ACR for three different categorization models (Zervides et al., 2022), and Ainiala’s revised criteria (Hawro et al., 2015; Li et al., 2019). Two studies that adhered to ACR criteria did not follow a standardized procedure (Engel et al., 2021) or use retrospective patient classification (Wegener et al., 2024). Yet, most studies adhere to ACR (Fragoso-Loyo et al., 2010; Jasiak-Zatoñska et al., 2022; Kammeyer et al., 2024; Lauvsnes et al., 2018; Lauvsnes et al., 2022; Noris-García et al., 2018; Portela et al., 2002; Schenatto et al., 2006; Wang et al., 2025; Yang et al., 2008), and most report the involvement of specialists in patient evaluation. In summary, most studies classified NPSLE using ACR criteria, and, in our opinion, this approach will aid in standardizing comparisons, given its potential for implementation in real-world settings.

Difficulties in directly comparing the biomarker across CNS versus PNS involvement arise from the inclusion of only CNS patients in some studies (Jasiak-Zatoñska et al., 2022; Lauvsnes et al., 2018; Portela et al., 2002; Trysberg et al., 2003), from classification that did not account for the biomarker in the analysis (Balajková et al., 2025; Kammeyer et al., 2024), or from not classifying neuropsychiatric manifestations (Hawro et al., 2015). Also, different grouping strategies for CNS versus PNS manifestations for analysis with biomarkers, such as comparing focal CNS, diffuse CNS, and PNS manifestations versus non-NPSLE (Engel et al., 2021), considering past and present neuropsychiatric symptoms (Lauvsnes et al., 2022), or only present symptoms (Fragoso-Loyo et al., 2010) into mutually exclusive CNS or PNS categories. For example, in Wegener et al.’s (2024) study, patients presenting with both CNS and PNS symptoms were excluded to enable comparison between the CNS and PNS groups (Wegener et al., 2024). Wang et al. (2025) categorized NPSLE patients according to past versus new/ongoing symptoms of focal CNS, diffuse CNS, and PNS manifestations (Wang et al., 2025). On the other hand, multiple studies compared the groups by individual manifestation against a control group (Noris-García et al., 2018; Schenatto et al., 2006; Yang et al., 2008; Zervides et al., 2022). Noris-García et al. (2018) classified patients based on their principal CNS or PNS manifestation, whether they have manifestations from both systems (Noris-García et al., 2018). This heterogeneity arises from naturally different manifestations across NPSLE cohorts, limited patient access, small sample sizes, and dilution of groups for comparison; consequently, it is difficult to reach consensus. However, the recommendation should always be to, as much as possible, differentiate types of neuropsychiatric manifestations and use these in statistical analyses to assess association with the biomarker.

On the other hand, SLE activity is assessed using validated indices, mainly the SLEDAI, and considering disease activity when studying potential biomarkers is of great importance. For example, Engel et al. (2021) and Trysberg et al. (2003) did not assess SLE activity for NPSLE (Engel et al., 2021; Trysberg et al., 2003), In other cases, despite the inclusion of the disease activity, no statistical approach was used to evaluate associations of the indices with NfL, GFAP (Kammeyer et al., 2024; Wegener et al., 2024), and S100B (Fragoso-Loyo et al., 2010; Schenatto et al., 2006) in NPSLE patients. Indirect associations between SLEDAI and S100B, and between SLEDAI and BDNF, can be examined in other studies (Noris-García et al., 2018; Yang et al., 2008). Importantly, statistical methods provide key information on the association between biomarkers and disease activity, at least in the entire SLE cohort (Balajková et al., 2025; Jasiak-Zatoñska et al., 2022; Lauvsnes et al., 2022; Zervides et al., 2022) or specifically in NPSLE patients (Hawro et al., 2015; Portela et al., 2002; Wang et al., 2025). In clinical biomarker research, activity indices enhance comparability across studies, support biomarker validation, and strengthen their potential diagnostic and prognostic value in NPSLE. Therefore, its use and analysis, preferably multivariable analyses, as an important NPSLE variable in biomarker research, is highly recommended.

Another important aspect is the study design; primarily cross-sectional studies were included, along with a few longitudinal studies for NfL and GFAP (Trysberg et al., 2003), S100B (Fragoso-Loyo et al., 2010; Yang et al., 2008), and TREM2 (Yang et al., 2008) in a small subset of patients. Interestingly, despite limitations in sample size, the results are promising. This encourages the need for further longitudinal studies of all biomarkers. Ideally, these studies should utilize a combination of candidate molecules to track changes over time and evaluate potential clinical applications for treatment, prognosis, and disease management.

Regarding biomarker studies, assessing discriminative capacity is of great importance; establishing cut-off values for further validation should also be a priority. For instance, cut-off values for diagnosis are scarcely reported. Serum NfL and GFAP have been investigated in diverse designs, yet most reports did not establish clear diagnostic thresholds, relying instead on mean comparisons or logistic regression models for NfL and GFAP (Balajková et al., 2025; Lauvsnes et al., 2022; Wegener et al., 2024; Zervides et al., 2022). Engel et al. (2021) evaluated the discriminating capacity of NfL with ROC curve analysis; however, cut-off values were not established (Engel et al., 2021). Conversely, Kammeyer et al. (2024) proposed the cut-off of NfL ≥ 6.8 pg/mL and GFAP ≥ 70.7 pg/mL to identify active major NPSLE. For serum S100B, while most of the studies did not assess cut-off values (Jasiak-Zatoñska et al., 2022; Lauvsnes et al., 2018; Portela et al., 2002; Yang et al., 2008), only Schenatto et al. (2006) and Noris-García et al. (2018) reported a cut-off point ≥ 0.125 ng/mL to differentiate NPSLE vs. non-NPSLE (Schenatto et al., 2006), and 0.022 ng/mL to differentiate peripheral polyneuropathy (Noris-García et al., 2018), respectively. Additionally, Fragoso-Loyo et al. (2010) reported that the S100B positivity was defined as values exceeding 3 standard deviations (SD) above the mean levels in the control group (Fragoso-Loyo et al., 2010). However, none of these results are directly related to interpreting and further applying this knowledge in clinical practice. This inconsistency underscores the lack of standardized criteria for biomarker validation in NPSLE. Recently, Wang et al. (2025) established a serum sTREM2 cut-off of ≥ 755.0 pg/mL to discriminate NPSLE and ongoing neuropsychiatric events (Wang et al., 2025). Lower reference range limit of NSE (8.22 μg/L) was used as a cut-off value to distinguish between NPSLE and non-NPSLE (Hawro et al., 2015). Unique studies on these biomarkers in NPSLE suggest their diagnostic potential. To conclude, this heterogeneity can be addressed by performing the necessary statistical analyses to assess the biomarkers’ discriminative capacity (e.g., ROC curve analysis) and by establishing cut-off values that maximize sensitivity and specificity. Such efforts would enable comparative analyses across studies, thereby strengthening the scientific evidence for the clinical use of these biomarkers and improving the management of patients with NPSLE.

The limitations extend to technical aspects of biomarker quantification. ELISA, digital immunoassays, multiplex bead assays, and chemiluminescent platforms were used inconsistently across studies, which may account for differences in reported units. In addition, several studies log-transformed levels, for example, for NfL (Engel et al., 2021; Zervides et al., 2022), while others retained the original units for the same biomarker (Balajková et al., 2025; Engel et al., 2021; Kammeyer et al., 2024; Lauvsnes et al., 2022; Trysberg et al., 2003; Wegener et al., 2024). This limits comparability and standardization of cut-off values (see Tables 1–3). Another source of bias is differences between samples: while most studies quantified molecules in serum, a few used plasma (Lauvsnes et al., 2022; Zervides et al., 2022), and one used both, with a later mathematical conversion from serum to plasma (Kammeyer et al., 2024). Such methodological diversity introduces variability in sensitivity and specificity, making cross-study synthesis problematic. As most studies focus on serum, this would be the best approach.

Taken together, these limitations underscore the urgent need for harmonized classification criteria, neuropsychiatric symptom categorization and analysis, disease activity assessment, standardized biomarker quantification methods, and longitudinal validation in larger, well-characterized cohorts that reflect real-world clinical scenarios. Also, statistical analysis should focus on multivariate methods, seeking predictive models and evaluating discriminative capacity and cut-off values to guide future clinical applications. These aspects are important for translating neurological damage-specific biomarkers into clinical practice. A reflection in this regard is needed for further study proposals.

## 5 Conclusion and perspective of translational application

Peripheral biomarkers of neuronal damage represent a promising but still evolving field in the study of NPSLE. Among them, NfL has emerged as the most consistent reported, showing strong potential to reflect central nervous system involvement. However, heterogeneity in patient’s classification, variability in control group selection, and the broad spectrum of clinical manifestations continue to limit the comparability of findings across studies. GFAP, although valuable, has not consistently outperformed NfL. At the same time, S100B has yielded encouraging results in serum analyses but remains challenged by limited specificity and extracerebral expression, underscoring the need for methodological standardization. Other molecules, such as UCHL-1 and BDNF, appear less promising in this context, whereas NSE and sTREM2, though scarcely studied, seem promising neurological damage-specific biomarkers.

Integrating these findings underscores the significant potential of applying peripheral biomarkers in clinical settings, which could greatly enhance clinical decision-making. This approach is particularly valuable for differentiating active neuroinflammation and effectively predicting and addressing neuropsychiatric symptoms and treatments. Specifically, neurofilament light chain (NfL) serves as a reliable indicator of axonal injury, while glial fibrillary acidic protein (GFAP) provides insight into astrocytic damage; both are fundamental to understanding the pathophysiology of neuropsychiatric systemic lupus erythematosus (NPSLE). Furthermore, while S100B is linked to neuroinflammation and neuronal injury, its expression outside the brain raises concerns about its specificity, necessitating careful interpretation. NSE and sTREM2 have been proposed as emerging biomarkers, relevant to neuronal and microglial biology, respectively. These biomarkers rise during active phases and decline in inactive states, highlighting their potential to differentiate neuroinflammatory activity. Although longitudinal studies remain scarce and often involve small cohorts, the findings are encouraging and suggest utility for monitoring treatment response and disease progression in such a heterogeneous condition. Moreover, the few available multivariate analyses indicate that combining these biomarkers with other biomarkers and clinical indices, such as SLEDAI, may enhance diagnostic precision and prognostic value. Future approaches may benefit from multivariate analyses that combine two or more biomarkers into diagnostic panels, potentially improving diagnostic accuracy and patient follow-up.

We highly encourage research on neurological damage-specific biomarkers, using classification of NPSLE and well-characterized cohorts by neuropsychiatric symptom types according to ACR criteria, disease activity assessment; longitudinal validation in larger, preferably assessing important clinical and laboratory variables that reflect real-world clinical scenarios, and importantly, the corresponding analysis with the biomarker levels in serum. Also, multivariate analytical methods, predictive models, and evaluation of the discriminative capacity of multiple biomarkers would have a greater impact. These aspects are important for translating neurological damage-specific biomarkers into clinical practice. Validated biomarker panels hold the key to translating molecular insights into meaningful clinical management of NPSLE. Therefore, these emerging biomarkers in neurodegenerative diseases warrant further investigation, particularly with well-characterized NPSLE phenotypes.
